# Left-censored dementia incidences in estimating cohort effects

**DOI:** 10.1007/s10985-020-09505-1

**Published:** 2020-09-12

**Authors:** Rafael Weißbach, Yongdai Kim, Achim Dörre, Anne Fink, Gabriele Doblhammer

**Affiliations:** 1 Chair in Statistics and Econometrics, Faculty for Economic and Social Sciences, University of Rostock, 18051 Rostock, Germany; 2 Department of Statistics, Seoul National University, Seoul, Korea; 3 German Center for Neurodegenerative Diseases, Bonn, Germany; 4 Chair in Empirical Social Research/Demography, Faculty for Economic and Social Sciences, University of Rostock, Rostock, Germany

**Keywords:** Censoring, Conditional likelihood, Confidence interval, Dementia, Hazard rate

## Abstract

We estimate the dementia incidence hazard in Germany for the birth cohorts 1900 until 1954 from a simple sample of Germany’s largest health insurance company. Followed from 2004 to 2012, 36,000 uncensored dementia incidences are observed and further 200,000 right-censored insurants included. From a multiplicative hazard model we find a positive and linear trend in the dementia hazard over the cohorts. The main focus of the study is on 11,000 left-censored persons who have already suffered from the disease in 2004. After including the left-censored observations, the slope of the trend declines markedly due to Simpson’s paradox, left-censored persons are imbalanced between the cohorts. When including left-censoring, the dementia hazard increases differently for different ages, we consider omitted covariates to be the reason. For the standard errors from large sample theory, left-censoring requires an adjustment to the conditional information matrix equality.

## Introduction

When studying the incidence of dementia, it is necessary to acknowledge the age of a person, and useful to study the evolution over time (cohort effect) (Doblhammer et al. 2013; Wu et al. 2016). From data of the nine-year period 2004 until 2012, we observe, for the German population born between 1900 and 1954, the ages at which dementia is diagnosed. For insurants of Germany’s largest Health insurance, we drew a simple random sample of 250,000 persons in 2004. An insurant with dementia incidence before the study period, i.e. prior to 2004, is left-censored. Together with the 80% right-censored persons without dementia in 2013, double-censoring is the required missing data pattern (see e.g. Ren and Gu 1997; Cai and Cheng 2004; Kim et al. 2013; Dörre and Weißbach 2017; Shen and Chen 2018).

We estimate the effect of cohort, age and sex from the Health Claims Data (HCD), with the cohorts in decades as dummy variables. Given that our data are a random sample, covariates are random as well, and we maximize the likelihood, conditional on the covariates (CMLE). In order to derive consistency and asymptotic normality for double censoring, as Ren and Gu (1997) and Cai and Cheng (2004) do, we apply the results about M-estimation, however for a different model or criterion function. Effort is devoted to obtaining a uniform convergence of the criterion functions with Wald’s dominating condition, so that convergence of the criterion function translates into convergence of the maximizing arguments. Also, the Conditional Information Matrix Equality needs to be adjusted for left-censoring, in order to avoid the need for sandwich estimation for an M-estimator in order to calculate standard errors for the confidence intervals.

As can be expected, for the HCD, we find that standard errors dip when including the 11,000 left-censored insurants. The cohort effect is generally negative in the sense that the dementia hazard has increased over the decades. However, with left-censoring, the slope of that increase is smaller. Another finding is that including left-censored persons increases the incidence of dementia at younger ages and attenuates the increase in dementia with age. That dementia is slightly more likely for males than for females becomes almost irrelevant after including left-censoring.

## Population and model for age-at-dementia-incidence

**Fig. 1:**
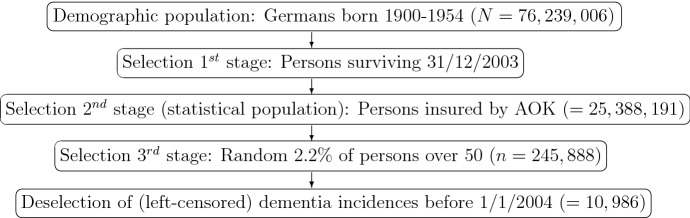
Data: trajectory from population to the right-censored persons via four stages of selecting persons (data for population size *N*: German Statistical Office (2004), without stillborn)

The population in the demographic sense are, basically Germans born between 1900 and 1954. We will not distinguish between different demarcation frontiers of Germany. As the statistical population, we will use insurance of a person by one German health insurer in 2004, and use its Health Claims Data (HCD). Note that health insurance is mandatory in Germany. The first three boxes in Fig. 1 depict the selection of people from the demographic to the statistical population. Our primary variable is age at dementia incidence and, roughly speaking, we wish to perform a lifetime data regression with the two covariates ‘cohort’, classified according to decades, and ‘gender’. As the age at dementia incidence is strictly positive, the theoretical simplicity of an additive model (see Kremer et al. 2014) is not appealing in demography, so that we model the effects multiplicative to the hazard as in Sect. III.1.4 of Andersen et al. (1993). An unspecified ‘baseline’ hazard $a_0(t)$, resulting in the semiparametric Cox-type model

$$\begin{aligned} a(t|{\mathbf {z}})= & {} a_0(t) e^{\mathbf {parameter}' {\mathbf {z}}}, \end{aligned}$$

would safeguard against model miss-specification. However, a widely acceptable weight function or smoothing parameter is out of sight in demography, whereas a Gompertz-baseline is standard.

### Definition 1

**Fig. 2:**
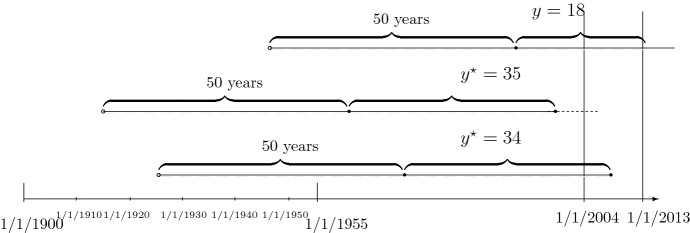
(Top: right-censored) Path for person born 1/1/1945 with dementia incidence after 1/1/2013, i.e. with $y=68-50=18$, $\delta =1$, ${\tilde{z}}_4=1$, ${\tilde{z}}_j=0$ for $j \in \{1,2,3,5\}$ (middle: left-censored) path for person born 1/1/1915 with dementia incidence 1/1/2000, i.e. with $y^{\star }=35$, $\delta =-1$, $y=89-50=39$, ${\tilde{z}}_2=1$, ${\tilde{z}}_j=0$ for $j \in \{1,3,4,5\}$ and death 1/1/2005 (bottom: uncensored) path for person born 1/1/1925 with dementia incidence 1/1/2009, i.e. with $y^{\star }=34$, $\delta =0$, ${\tilde{z}}_3=1$, ${\tilde{z}}_j=0$ for $j \in \{1,2,4,5\}$

The duration $Y^{\star }$ in years between the person’s 50th birthday (risk onset) and dementia incidence has a hazard rate, conditional on ${\mathbf {Z}}={\mathbf {z}}$,

$$\begin{aligned} a(t|{\mathbf {z}})= & {} a e^{\beta _1 t} e^{\varvec{\beta }_2' \tilde{{\mathbf {z}}}} e^{\beta _3 z^s} = e^{\varvec{\theta }'(1,t,{\mathbf {z}}')'}, \end{aligned}$$

where *t* (also) denotes the age (since the 50th birthday) and ${\mathbf {z}}:=(\tilde{{\mathbf {z}}}', z^s)'$. We have $\theta _1=\log a$. It is ${\tilde{z}}_1=1$ for a person born between 1900 and 1909, and zero if it is born in some other decade. It is ${\tilde{z}}_2=1$, ${\tilde{z}}_3=1$ or ${\tilde{z}}_4=1$ for a person who was born in the 1910s, the 1920s or the 1940s. It is ${\tilde{z}}_5=1$ for a person who was born between 1950 and 1954, the latest date possible for a person to become 50 years old, prior to the start of the study in 2004. (The thirties are the reference cohort.) The coding of cohorts is displayed in Table 1, and Fig. 2 displays (at the bottom) the coding for one uncensored person, i. e. with dementia incidence during the study period. The $z^s$ codes the sex (0=male; 1=female). We denote by $e^{\beta _1 t}$, or $\beta _1$, the age effect, and by $e^{\varvec{\beta }_2' \tilde{{\mathbf {z}}}}$, or $\varvec{\beta }_2$, the cohort effect. In short, the eight parameters, $\theta _k$, of the model are $\varvec{\theta }:=(\log a,\beta _1, \beta _{21}, \ldots , \beta _{25}, \beta _3)'$. We assume that the distribution of ${\mathbf {Z}}$ does not depend on $\varvec{\theta }$. $\square$

**Table 1:** Assigning coordinates of $\varvec{\beta }_2$ to the respective cohorts and corresponding indicator covariate $\tilde{{\mathbf {z}}}$

Cohort	Parameter	Dummy
1900–1909	$\beta _{21}$	${\tilde{z}}_{1} = 1$
1910–1919	$\beta _{22}$	${\tilde{z}}_{2} = 1$
1920–1929	$\beta _{23}$	${\tilde{z}}_{3} = 1$
1930–1939	–	Reference cohort
1940–1949	$\beta _{24}$	${\tilde{z}}_{4} = 1$
1950–1954	$\beta _{25}$	${\tilde{z}}_{5} = 1$

The cohort 1930–1939, being the largest, acts as reference cohort and therefore possesses no parameter

Dementia is very rare before the age of 50 (see Harvey et al. 2003; Ikejima et al. 2009) and we consider only persons aged 50 years and above in our study. Note also that according to the last representation of the model, it contains a time-dependent covariate. The combination of the constant baseline hazard *a* and age effect $e^{\beta _1 t}$ is the Gompertz distribution (see Sect. 22.8 Johnson et al. 1995), which is usual in the demography of age-dependent morbidity and death. For the other typical demographic baseline of a Weibull hazard, *t* would have to be replaced by $\log t$ (see Chap. 21 Johnson et al. 1994).

From the conditional hazard function $a(\cdot |{\mathbf {z}})$ of Definition 1, we require that $\beta _1 > 0$ and derive as the conditional cumulative hazard rate, density and CDF:

1 $$\begin{aligned} A(y^{\star }|{\mathbf {z}})= & {} \int _0^{y^{\star }} a(t|{\mathbf {z}}) dt = \frac{a}{\beta _1} e^{\varvec{\beta }_2' \tilde{{\mathbf {z}}} + \beta _3 z^s} \left( e^{\beta _1 y^{\star }} - 1\right) \nonumber \\ f(y^{\star }|{\mathbf {z}})= & {} a(y^{\star }|{\mathbf {z}}) \exp [-A(y^{\star }|{\mathbf {z}})] \nonumber \\ F(y^{\star }|{\mathbf {z}})= & {} 1 - \exp [-A(y^{\star }|{\mathbf {z}})] \end{aligned}$$

## Health claims data, censoring and criterion function

We use HCD from the Allgemeine Ortskrankenkasse (AOK), the largest public health insurance company in Germany. The claims data contain information about outpatient and inpatient diagnoses and treatments, on a quarterly basis, for each insured person, with at least one day of insurance coverage, regardless of whether they sought medical treatment or not. The data include information about sex, age, year of birth and date of exit (death or switch to another insurance company). All inpatient and outpatient diagnoses are coded in the International Statistical Classification of Diseases and Related Health Problems (ICD), revision 10, issued by the World Health Organization. For this study, the health insurance company drew a random sample of 250,000 persons with a follow-up until the end of 2012 given that the persons were insured in the first quarter of 2004. This corresponds to approximately 2.2% of the statistical population who was born before 1955 and has survived the year 2003 (see Sect. 2). Dementia is defined by the ICD-10 numbers G30, G310, G3182, G231, F00, F01, F02, F03 and F051 for which we exclusively consider outpatient diagnoses with the modifier verified, and discharge secondary diagnoses from the inpatient sector. We do not distinguish according to aetiology, and combine all ICD codes into one group named dementia. The method of diagnosis validation is laid out in Doblhammer et al. (2013) and it results in $n=245,888$ observations after data cleaning.

Let us consider the potential obstacles when applying Definition 1 to the HCD. Consider the dummy variable that codes the cohort, e.g. ${\tilde{Z}}_4$ for the 1940s (see again Table 1). Its parameter is the probability of selecting a person born in that decade from the insurants. The variable is ‘exogenous’ and will not disturb our inference to the *statistical* population (see again Fig. 1), as we will use the conditional likelihood. Inference to the *demographic* population will be considered in Sect. 5.2.

The typical obstacle to statistical inference for Definition 1 is that the duration $Y^{\star }$ maybe subject to right- or left-censoring. Occasionally, left-censored observations are deselected in the demographic literature, as depicted in the last box of Fig. 1, and we aim in this study to assess the consequences thereof. Let us derive the censoring notation. For each person, we record its year and month of *birth*, but the year and month of *death* is only recorded if it is in the study period between 1/1/2004 and 31/12/2012. We denote the age at the start of the study period on 1/1/2004, given in months since 50th birthday, by *L*. Birth and death are assumed to occur in the middle of a month. For each person, we observe the age in months at the time of dementia incidence, date of death, loss to follow-up or end of study. We assume an onset of dementia risk at the age of 50 and denote the subsequent time as *Y*. We attribute the diagnosis to the *middle* of a quarter, and a loss to follow-up at the *end* of a quarter. A censoring indicator $\Delta$ is 0, i.e. $Y^{\star }$ is uncensored, if the dementia incidence occurs in the study period. It is $\Delta =1$, i.e. $Y^{\star }$ is right-censored, if (i) the incidence is past the study period (i.e. after the forth quarter of 2012 (Q4/2012)), (ii) a patient dies without having had dementia, or (iii) is lost to follow-up. The censoring is $\Delta =-1$, and $Y^{\star }$ is left-censored, if the incidence has been prior to the study period, i.e. before Q3/2004. It is also assumed that a dementia diagnosis in Q1/2004 and Q2/2004 is a prevalent case, i.e. a left-censored observation (see Table 2).

**Table 2:** Observed ages-at-dementia and censoring information: numbers of persons

Left-censored	Uncensored	Right-censored
10,986 (4.47%)	35,920 (14.61%)	198,982 (80.92%)

Altogether:

$$\begin{aligned} Y := {\left\{ \begin{array}{ll} Y^{\star } \;\text {(age at dementia diagnosis - 50)} &{} \text {for} \; L \le Y^{\star } \le R \quad (\Delta :=0)\\ L \;\text { (age at beginning of study period -50)} &{} \text {for}\; Y^{\star }< L \quad (\Delta :=-1) \\ R \;\text { (age at event (i)-(iii) minus 50)} &{} \text {for} \; R < Y^{\star } \quad (\Delta :=1) \\ \end{array}\right. } \end{aligned}$$

Note that although the study period is fixed, entrance is individually different, depending on the birthday. We do not (and do not need to) model the birthday, as we can leave the joint distribution for (*L*, *R*) unspecified, apart from $R>L$. We denote its parameter by $\varvec{\theta }_{\Delta }$. As a consequence, the censoring indicator $\Delta$ is random. We will see that $\Delta$ is *endogenous*, that is its distribution depends on $\varvec{\theta }$. As specified for ${\tilde{Z}}_4$ earlier, the entire covariate ${\mathbf {Z}}$ is random due to sampling and *exogenous*. The values *y*, $\delta$ and ${\mathbf {z}}$ can obviously be calculated with the definitions from Sect. 2 for each person. Figure 2 displays the coding for a right- and a left-censored person (top and middle), i. e. with dementia incidence outside the study period.

We need the distribution of $(Y,\Delta ,{\mathbf {Z}})$ when defining the criterion function. To derive the density, we commence with left-censoring and assume $Y^{\star }$ and *L* to be independent. The age at the beginning of the study period, *L*, is also assumed to be independent of ${\mathbf {Z}}$ and we denote its density and CDF as $f_L(\cdot )$ and $F_L(\cdot )$. We observe

2 $$\begin{aligned} Y:=\max \{ Y^{\star },L \} \quad \text {and} \quad \Delta :=\mathbb {1}_{\{Y^{\star }\ge L\}} - 1. \end{aligned}$$

with density

3 $$\begin{aligned} \quad f_{Y,\Delta |{\mathbf {Z}}}(y, \delta |{\mathbf {z}}) = f(y|{\mathbf {z}})^{1 + \delta } F(y|{\mathbf {z}})^{-\delta } f_L(y)^{-\delta } F_L(y)^{ 1+ \delta }. \end{aligned}$$

Note that the conditional density $f(\cdot |\cdot )$ and CDF $F(\cdot |\cdot )$ are those of the ‘latent’ $Y^{\star }$. Right-censoring instead of left-censoring is similar, only with $F(y|{\mathbf {z}})$ replacing $1-F(y|{\mathbf {z}})$, and $1- F_R(y)$ replacing $F_L(y)$. Of course, with dementia the residual lifetime will be reduced so that, for the right-censoring cause (ii), age-at-death and age-at-dementia will not be stochastically independent. However, we think it is non-informative because we are not interested in mortality, but in morbidity and that death has an impact on the—still conceptionally existing—time until dementia is not plausible. The conditional density under double-censoring is easily derived (see Proposition 1 in Dörre and Weißbach (2017) or Formula (3) in Kim et al. 2013).

As the first step towards the criterion function, recall that the distribution of ${\mathbf {Z}}$ does not depend on $\varvec{\theta }$. The parameter of the covariate is nuisance, so ‘conditioning’ applies (see Kalbfleisch and Sprott 1970; Reid 1995). For the bivariate dependent variable $(Y, \Delta )$, the conditional likelihood method is appropriate for estimating the parameter vector $(\varvec{\theta },\varvec{\theta }_{\Delta })$. Note that due to endogeneity, it is not possible to separately relate $\varvec{\theta }$ to *Y* and $\varvec{\theta }_{\Delta }$ to $\Delta$. Also note that the categorical scale of the second dependent variable, $\Delta$, is not an obstacle, as more importantly, $\varvec{\theta }_{\Delta }$ is on a continuous scale.

Ultimately, we want to restrict attention to $\varvec{\theta }$. As the distribution of *L* is unconnected to the parameter $\varvec{\theta }$, the third and forth factors of (3) will not influence the point estimate found by maximization with respect to (wrt) $\varvec{\theta }$, as can be seen from the usual logarithmic transformation. The same is true for *R*. The impact of $\varvec{\theta }_{\Delta }$ on the standard errors is studied in the next Sect. 4. Note already that $\varvec{\theta }$ can be gained from the conditional model by the smooth function $\varvec{\theta }:=g(\varvec{\theta },\varvec{\theta }_{\Delta })$. The conditional likelihood, denoted by $\ell ^c(\varvec{\theta },\varvec{\theta }_{\Delta })$, is the product over (3) (amended by right-censoring), as indeed $(Y^{\star }_i, L_i, R_i, {\mathbf {Z}}_i')$—and hence $((Y_i, \Delta _i), {\mathbf {Z}}_i')$—are independent and identically distributed. We now collect all factors in $\ell ^c(\varvec{\theta },\varvec{\theta }_{\Delta })$ that contain $\varvec{\theta }$ and define

4 $$\begin{aligned} \Psi [(y_i, \delta _i), {\mathbf {z}}_i; \varvec{\theta }]:= & {} \mathbb {1}_{\{\delta _i=0\}} \log [f(y_i|{\mathbf {z}}_i)] + \mathbb {1}_{\{\delta _i=-1\}} \log [F(y_i|{\mathbf {z}}_i)] \nonumber \\&+ \mathbb {1}_{\{\delta _i=1\}} \log [1 - F(y_i|{\mathbf {z}}_i)]. \end{aligned}$$

Note that due to the last summand, we need observations for *all* persons. We cannot expect a low-dimensional sufficient statistic as is occasionally the case for only right-censored survival data. We define the exponential of our *criterion function* as

5 $$\begin{aligned} \prod _{i=1}^n \exp \{\Psi [(Y_i, \Delta _i), {\mathbf {Z}}_i; \varvec{\theta }]\}. \end{aligned}$$

The maximizing argument is denoted by $\hat{\varvec{\theta }}$ and numerically determined. An initial value must avoid negatively infinite log-conditional-likelihood values. Specifically, we start from a model with only the 35, 920 uncensored observations and without ${\mathbf {Z}}$, i.e. with $\varvec{\beta }_2={\mathbf {0}}$ and $\beta _3=0$. The closed-form estimate for $(a, \beta _1)'$ is then $(0.22 \times 10^{-3}, 0.152)'$. Using now the covariates, the logarithmic value for (5) has a numerically maximal value of $-425,851$ at

$$\begin{aligned} \widehat{\varvec{\theta }}_{uncens.}'=(0.459 \times 10^{-3},0.234, -5.016, -3.545, -1.788, 2.365, 4.815, -0.155). \end{aligned}$$

Including now censored observations, the logarithmic (5) has for all $n=245,888$ observations a numerical maximum of $-191,444$. The adequacy of the numerical maximizations were verified ex post for convergence. The resulting point estimates are given in Table 3 (as the two rows ‘Definition 1’, with and without the left-censored observations) and will be discussed in Sect. 5, together with standard errors derived in the next section.

## Statistical inference

Let us here study the implication of left-censoring for estimator consistency and normality, the latter with consequences for the confidence intervals. Along with the nuisance parameter for censoring, $\varvec{\theta }_{\Delta }$, the distribution of the random (multivariate) covariate also, has a parameter which we do not denote explicitly. Roughly speaking, studying the asymptotic normality of a Maximum Likelihood Estimator (MLE) for *all* parameters enables deferring the normality of the estimator for $\varvec{\theta }$, even when only maximizing (5).

In more detail, as stated, we aim at disposing of the parameter of the (exogenous) covariate by conditioning, i.e. by factorizing the likelihood. As censoring is endogenous, instead of conditioning, we aim at disposing of the $\varvec{\theta }_{\Delta }$ by the virtue of fact that differentiation of $\Psi (\cdot )$, and of the logarithmic unconditional density of $[(Y_i,\Delta _i),{\mathbf {Z}}_i']$ wrt to $\varvec{\theta }$, are equal. To see this, factorize the latter unconditional density into (3) and the marginal density of ${\mathbf {Z}}_i$. After taking the logarithm and differentiating, the marginal density of ${\mathbf {Z}}_i$ and the distribution of $(L_i,R_i)$ vanish, as both are assumed not be depend on $\varvec{\theta }$.

Note already that, for the derivation of asymptotic confidence intervals, including standard errors, arguments will be needed that prevent the use of the entire distribution, which would again include the covariate parameter and $\varvec{\theta }_{\Delta }$.

In order to establish the asymptotic normality of the estimator, in Sect. 4.2, we will use a Taylor expansion of the score equation. An important requirement on several occasions will be the consistency of $\hat{\varvec{\theta }}$ (maximizing (5)). There are several sets of assumptions underlying such a proof (see e.g. Property 8.1 in Gouriéroux and Monfort 1995a). The main idea behind Wald’s dominating conditions (6) is to ensure that the convergence of the criterion function (as a sequence in *n*) will be uniform (as function of $\varvec{\theta }$). This will in turn ensure the convergence of the maximizing argument, $\hat{\varvec{\theta }}$, to converge to the true parameter $\varvec{\theta }_0$ for $Y^{\star }$ (conditional on ${\mathbf {Z}}={\mathbf {z}}$).

### Wald’s dominating condition

Even though we want to cover double-censored durations, we commence with uncensored observations. And, for simplicity of the argument, we start without covariates. Hence, the criterion function (5) reduces to the likelihood, and for the MLE, we verify Wald’s D conditions. Of the Wald-conditions, especially condition D3 is cumbersome, namely to find an integrable positive function $h(y^{\star })$ that dominates the likelihood ratio:

6 $$\begin{aligned}&\left| \log \frac{f(y^{\star }; \varvec{\theta })}{f(y^{\star }; \varvec{\theta }_0)} \right| \le h(y^{\star }) \quad \forall \; \varvec{\theta } \in \varvec{\Theta } \; \text {(being compact)} \nonumber \\&\text {with} \quad \int _{\mathbb {R}^+} h(y^{\star }) f(y^{\star }; \varvec{\theta }_0) dy^{\star } < \infty \end{aligned}$$

Here $f(y^{\star }; \varvec{\theta })$ is synonymous for $f(y^{\star }|{\mathbf {z}})$ in (1). The idea is to set $h(y^{\star })$ as the upper bound to the left in the first inequality of (6)—wrt to $\varvec{\theta }$—and to show integrability, wrt to $y^{\star }$.

Let us set $\Theta = [\varepsilon ;\frac{1}{\varepsilon }]$, for some small $\varepsilon >0$ and even ignore the age-effect up to this point, i.e. set $\beta _1=0$.

#### Lemma 1

For independent $Y_1^{\star }, \ldots , Y_n^{\star } \sim Exp(a_0)$ and $a_0 \in (\varepsilon ;\frac{1}{\varepsilon })$, Wald’s D3-condition (6) holds.

**Fig. 3:**
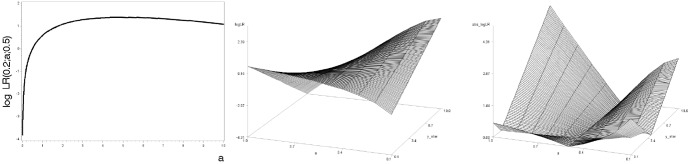
Left: log likelihood ratio (7) for $y^{\star }=0.2$ and $a_0=0.5$, middle: log likelihood ratio (7) for $a_0=0.5$, right: absolute of log likelihood ratio for $a_0=0.5$

The proof stems from the following graphical arguments. Obviously, in the $y^{\star }$-direction, the log-likelihood ratio

7 $$\begin{aligned} \log LR(y^{\star }, a, a_0):= \log \frac{f(y^{\star }; a)}{f(y^{\star }; a_0)} = \log \frac{a}{a_0} - (a - a_0) y^{\star } \end{aligned}$$

is linear. It increases for $a<a_0$ and decreases for $a_0<a$. In both cases, the slope decreases (in absolute terms) as *a* approaches the true parameter $a_0$. For $a=a_0$ the function is constant. More important is the direction of argument *a*, for which $\log LR(y^{\star }, a, a_0)$ is concave (see Fig. 3 (left)). As a function of both arguments, the ratio has the shape of a pear leaf (Fig. 3 (middle)). The log likelihood ratio is concave in *a* with local minima at the edges of the parameter space, $\{\varepsilon ;1/\varepsilon \}$. If the function were negative, these would be the only potential maxima of the absolute value. Unfortunately, this is not the case, as Lemma 4 in “Appendix A” exhibits.

**Fig. 4:**
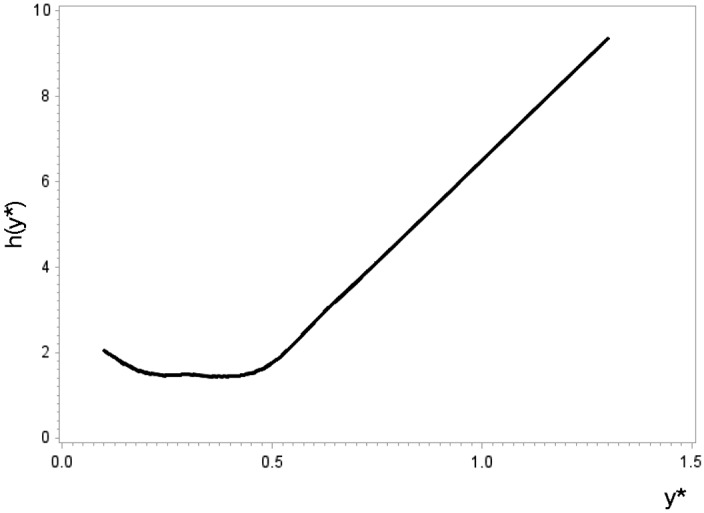
Bound $$h(y^{\star })$$ for $$a_0=0.5$$

Hence, function

$$\begin{aligned} \left| \log LR(y^{\star }, a, a_0) \right| = | \log \frac{a}{a_0} - (a - a_0) y^{\star } | \end{aligned}$$

has its maximum either in $a= \varepsilon$, in $a=1/\varepsilon$ or in $a=1/y^{\star }$. Figure 3 (right) displays the ‘used-handkerchief shape’ that $\log LR(y^{\star }, a, a_0)$ exhibits as a function of *a* and $y^{\star }$. The function $h(y^{\star })$ is composed as maximum over the only three candidates $\varepsilon$, $1/y^{\star }$ and $1/\varepsilon$. The analytical version of the proof is in “Appendix A”. Graphically, one considers the three one-dimensional functions through the three-dimensional room depicted in Fig. 3 (right) as candidates. For the first two candidates, whatever $y^{\star }$, the maximum is at the same *a*, namely on the edge (on the room’s left and right wall). These candidate functions are parallel. This is not true for the third function because the maxima are at $a=1/y^{\star }$ in the parameter space. (It proceeds in a curve through the room.) Now imagine the two-dimensional vertical plane spanned by the $y^{\star }$-axis and the axis of the log-likelihood (i.e. the left wall of a room you enter). And imagine a projection of the three function graphs on that plane (as shadows on the left wall near a light source on the right wall), the upper hull in this picture is the graph of $h(\cdot )$. For the example $a_0=0.5$, Fig. 4 depicts $h(\cdot )$ and suggests that one linear edge extremum quickly dominates the other two candidates.

As consequence, the second half of condition (6) is fulfilled as it is proportional to the expectation of an exponential distribution, being $1/a_0$ and hence finite, due to the compact support of the parameter space.

The aim is now to include the left-censoring. But then, the criterion function will not be the likelihood, but only a factor thereof. It is the product over

$$\begin{aligned} f_{Y,\Delta }(y, \delta ;a) \propto f(y;a)^{1+ \delta } F(y;a)^{-\delta } \end{aligned}$$

so that without right-censoring and covariates, (4) becomes

$$\begin{aligned} \Psi _L(y,\delta ;a):= (1+ \delta ) \log f(y;a) - \delta \log F(y;a). \end{aligned}$$

There is an analogous criterion to Wald’s D3-condition (6) for M-estimation (see Sect. 24.2.3, condition C2’ in Gouriéroux and Monfort (1995b), originally Theorem 2 from Jennrich 1969). For the proof of the following Lemma, see 1.

#### Lemma 2

Assume $Y_1^{\star }, \ldots , Y_n^{\star } \sim Exp(a_0)$ and $L_1, \ldots , L_n \sim F_L(\cdot )$ to be all independent and $0<P(Y^{\star }<L)<1$. For *Y* and $\Delta$ as in (2), there is a function $h_L(y,\delta )$ such that

$$\begin{aligned} | \Psi _L(y,\delta ;a) | \le h_L(y,\delta ) \quad \forall a \in \left[ \varepsilon , \frac{1}{\varepsilon }\right] \end{aligned}$$

with

8 $$\begin{aligned} \sum _{\delta \in \{-1,0\}} \int _{\mathbb {R}^+} h_L(y,\delta ) f_{Y,\Delta }(y,\delta ; a_0) dy < \infty . \end{aligned}$$

In contrast to the method of proof in Kremer et al. (2014), the method here easily extends to double-censored observations. The sum (8) then has three summands. And in the third summand, $h_L(y,1)=\frac{1}{\varepsilon }y$ is easily found by the linearity of *ay* wrt *a*. The resulting integral is then again proportional to the expectation of $Y^{\star }$, given that right-censoring is neither impossible nor sure. The Lemma also carries over to covariates, as we consider the conditional densities. The aim is also to include the age-effect. The parameter space now has two dimensions, $\beta _1$ being between a small and a large positive real value and the log-likelihood ratio becomes (see Definition 1 and (1))

$$\begin{aligned} \log \frac{f[y^{\star }; (a,\beta _1)]}{f[y^{\star }; (a_0,\beta _{10})]}= & {} \log \frac{a}{a_0} + (\beta _1 - \beta _{10})y^{\star } - \frac{a}{\beta _1}\left( e^{\beta _1 y^{\star }}-1\right) \\&+ \frac{a_0}{\beta _{10}}\left( e^{\beta _{10} y^{\star }}-1\right) \end{aligned}$$

as $A(y^{\star })=\frac{a}{\beta _1}\left( e^{\beta _1 y^{\star }}-1\right)$ and $\log f[y^{\star }; (a,\beta _1)]=\log a(y^{\star }) - A(y^{\star })$, $\log a(y^{\star })=\log a + \beta _1 y^{\star }$. Now the second half of (6) does not simplify to an expectation, but interchanging the integral with the sum, the summands are either limited because of the density property, because of the finite expectation, or because $e^{\beta _1 y^{\star }}$ can be absorbed into the exponential function of the density to form a new Gompertz distribution’s density. Taking maxima over the parameter space does not hinder this, as the parameter space is bounded and all functions are continuous in the parameters.

### Standard error for $\hat{\varvec{\theta }}$

The maximum of (5) also maximizes $\ell ^c(\varvec{\theta },\varvec{\theta }_{\Delta })$ wrt to the first argument. It even maximizes the likelihood wrt to $\varvec{\theta }$, as we assume the distribution of ${\mathbf {Z}}$ not to depend on $\varvec{\theta }$. Neither $\varvec{\theta }_{\Delta }$ nor the parameters of the covariate ${\mathbf {Z}}$ are of concern for the point estimate. The asymptotic standard error of an MLE, classically builds upon the unconditional expectation of the squared gradient of the logarithmic density for one observation, namely the Fisher information matrix. Such expectation wrt the joint distribution of $((Y_i, \Delta _i), {\mathbf {Z}}_i')$ will add $\varvec{\theta }_{\Delta }$ and the covariate parameters into the expression. Roughly speaking, we can partition the Fisher information matrix into blocks, where the upper left block is for $\varvec{\theta }$, and then, on the block-diagonal a block for $\varvec{\theta }_{\Delta }$ follows, and the lower right block is for the covariate parameters. The arguments for point estimation also let conclude that the off-diagonal block matrices will all be zero. Classically, the standard errors for the parameters are deduced from the inverse of the Fisher information. Due to the Schur complement (see e.g. Section A.2.2.d in Gouriéroux and Monfort 1995b), only the inverse of the upper-left block must be inverted to achieve a standard error of $\varvec{\theta }$. Standard errors can be estimated with the observed Fisher information.

Let us now be more specific and denote by $E_0$ the expectation wrt $(Y,\Delta )$, conditional on ${\mathbf {Z}}$, by $E_{{\mathbf {Z}}}$ the expectation wrt to the marginal distribution of ${\mathbf {Z}}$. Define, with finally unconditional expectations

$$\begin{aligned} \varvec{{\mathcal {J}}}:= & {} E_{{\mathbf {Z}}} E_0 \left( - \frac{\partial ^2 \Psi [(Y, \Delta ), {\mathbf {Z}}; \varvec{\theta }_0]}{\partial \varvec{\theta } \partial \varvec{\theta }' }\right) \\ \varvec{{\mathcal {I}}}:= & {} E_{{\mathbf {Z}}} E_0 \left( \frac{\partial \Psi [(Y, \Delta ), {\mathbf {Z}}; \varvec{\theta }_0]}{\partial \varvec{\theta } }\frac{\partial \Psi [(Y,\Delta ), {\mathbf {Z}}; \varvec{\theta }_0]}{\partial \varvec{\theta }' } \right) . \end{aligned}$$

#### Theorem 1

For the maximizing argument $\hat{\varvec{\theta }}$ of (5), it holds for a compact subspace for $\varvec{\theta }_0$ in $(\mathbb {R}^+)^2 \times \mathbb {R}^6$:

$$\begin{aligned} \sqrt{n}\left( \hat{\varvec{\theta }} - \varvec{\theta }_0\right) {\mathop {\longrightarrow }\limits ^{d}} N_8({\mathbf {0}}, \varvec{{\mathcal {J}}}^{-1} \varvec{{\mathcal {I}}} \varvec{{\mathcal {J}}}^{-1}) \end{aligned}$$

#### Proof

Denote the column vector

$$\begin{aligned} {\mathbf {U}}(\varvec{\theta }):= \nabla _{\varvec{\theta }} \log \ell ^c(\varvec{\theta },\varvec{\theta }_{\Delta })=\nabla _{\varvec{\theta }} \sum _{i=1}^n \Psi [(Y_i, \Delta _i), {\mathbf {Z}}_i; \varvec{\theta }] \end{aligned}$$

and perform a multivariate quadratic Taylor expansion thereof, evaluated at the maximizing argument and expanded around the true parameter value for each of the eight coordinates:

$$\begin{aligned} 0= & {} \frac{1}{\sqrt{n}}U^k(\hat{\varvec{\theta }}) = \frac{1}{\sqrt{n}} {\mathbf {U}}^k(\varvec{\theta }_0) - \frac{1}{n} \nabla _{\varvec{\theta }}U^k(\varvec{\theta }_0)' \sqrt{n}(\hat{\varvec{\theta }} - \varvec{\theta }_0) \\&+ \sqrt{n} (\hat{\varvec{\theta }} - \varvec{\theta }_0)'\frac{1}{2n} \nabla ^2_{\varvec{\theta }}U^k(\varvec{\theta }^{*})(\hat{\varvec{\theta }} - \varvec{\theta }_0) \end{aligned}$$

Here $\nabla ^2_{\varvec{\theta }} U^k(\varvec{\theta }^{*})$ denotes the Hessian matrix and $\varvec{\theta }^{*}$ is a point on the line between $\hat{\varvec{\theta }}$ and $\varvec{\theta }_0$. The last summand is asymptotically negligible by Slutzky’s Lemma, for three reasons: (i) Its second factor, the Hessian (divided by *n*), can be shown to be bounded at $\varvec{\theta }_0$ by the usual arguments of continuous functions on compact support and because $\varvec{\theta }^{*}$ will converge to $\varvec{\theta }_0$ because $\hat{\varvec{\theta }}$ is consistent. (ii) Its last factor converges to zero, as $\hat{\varvec{\theta }}$ is consistent. (iii) Its first factor (including $\sqrt{n}$) converges weakly. The entire last summand is dropped in the following analysis. In a more precise version of the proof, one applies Theorem 10.1 of Billingsley (1961).

The first summand ${\mathbf {U}}^k(\varvec{\theta }_0)/\sqrt{n}$ is now a sum of iid random variables and will be asymptotically normal with mean zero and variance-covariance matrix $\varvec{{\mathcal {I}}}$, due to the CLT by the usual arguments. Subtract the second summand, and multiply the equation with the inverse of $\nabla ^2_{\varvec{\theta }} U^k(\varvec{\theta }^{*})/n$. It becomes a first factor on the right side and can be replaced with its deterministic matrix limit, namely $\varvec{{\mathcal {J}}}^{-1}$, by the LLN. (We refrain from verifying a sufficient assumption such existence of moments or differentiability for the characteristic function of $\nabla _{\varvec{\theta }} \Psi [(Y_i, \Delta _i), {\mathbf {Z}}_i; \varvec{\theta }]$.) We will also not verify that the model is identified. $\square$

For a conditional likelihood $\ell ^c(\varvec{\theta },\varvec{\theta }_{\Delta })$, the ‘Conditional Information Matrix Equality (CIME)’ follows, and for our criterion function, i.e. the logarithm of (5), a similar equation holds for $\varvec{\theta }$.

#### Lemma 3

$$\varvec{{\mathcal {I}}} = \varvec{{\mathcal {J}}}$$.

The proof uses elementary analysis and is given in  “Appendix B”. As a consequence $\varvec{{\mathcal {J}}}^{-1} \varvec{{\mathcal {I}}} \varvec{{\mathcal {J}}}^{-1}=\varvec{{\mathcal {J}}}^{-1}$. By Theorem 1, the asymptotic standard errors can be derived now as square roots of the diagonal elements from $\varvec{{\mathcal {J}}}^{-1}$ and consistently estimated from

9 $$\begin{aligned} \hat{\varvec{{\mathcal {J}}}}:=\frac{1}{n}\sum _{i=1}^n - \frac{\partial ^2 \Psi [(y_i, \delta _i),{\mathbf {z}}_i; \hat{\varvec{\theta }}]}{\partial \varvec{\theta } \partial \varvec{\theta }'}. \end{aligned}$$

There are only minor numerical considerations when estimating the standard errors for the eight-dimensional function of Definition 1 with a Newton algorithm. The resulting standard errors are given in the rows ‘Definition 1’ of Table 3 (in brackets below the point estimates).

## Data analysis

### For the statistical population

Let us here study the implications of left-censoring on the estimates from HCD, especially on the confidence intervals. We now study the implication of deselection for the inference from the sample (3rd stage selection) onto the statistical population (2nd stage selection) (see Fig. 1). The rows ‘Definition 1’ in Table 3 summarize the inference drawn from the sample for $\varvec{\theta }$ in the statistical population of Germans born between 1900 and 1954 and insured by the AOK in 2004. The results enable an assessment of excluding or including left-censored observations (see third to fifth boxes in Fig. 1). As a first general finding, by including left-censoring, confidence intervals become narrower for almost all parameters, as to be expected, with the exception of *a*. Overall, given the interpretation of the standard error as half of a half confidence interval by Theorem 1, no small sample size argument needs to be taken into consideration and almost all effects are statistically significant in the sequel.

Before we compare point estimates and standard errors for the model of Definition 1, we fit two smaller preliminary models for dementia incidence to the data. Both models neglect the gender effect, i.e. generally set $\beta _3=0$. In one model, we neglect the age effect, i e. set $\beta _1=0$, and in the other model the cohort effect is neglected, i.e. we set $\varvec{\beta }_2=0$. Of course we are convinced that both effects exist, but still want to build a model by forward selection of covariates.

**Table 3:** Estimates for model of Definition 1 subject to right-censoring (top) and double-censoring (bottom)

			Age-effect	1900–1909	1910–1919	1920–1929	1940–1949	1950–1959	Sex-effect
		${\hat{a}}$ ($\times 10^3$)	${\hat{\beta }}_1$	${\hat{\beta }}_{21}$	${\hat{\beta }}_{22}$	${\hat{\beta }}_{23}$	${\hat{\beta }}_{24}$	${\hat{\beta }}_{25}$	${\hat{\beta }}_3$
Number of persons in sample (by birth decade)				1698	15452	51720	64197	27663	
Excluding left-censored persons	Only cohort-effect	4.997	–	0.651	0.881	0.702	− 0.755	− 0.994	–
		[4.90, 5.10]		[0.56, 0.74]	[0.85, 0.91]	[0.68, 0.73]	[-0.80, − 0.71]	[-1.08, − 0.91]	
	Only age-effect	0.221	0.152	–	–	–	–	–	–
		[0.213, 0.229]	[0.150, 0.154]						
	Cox-type	–	–	− 3.767	− 2.348	− 1.103	1.222	2.667	− 0.092
		–	–	[− 3.88, − 3.66]	[− 2.40, − 2.30]	[− 1.14, − 1.07]	[1.17, 1.28]	[2.55, 2.79]	[− 0.11, − 0.07]
	Definition 1	0.058	0.226	− 3.476	− 2.035	− 0.816	0.917	2.084	− 0.087
		[0.056, 0.060]	[0.224, 0.228]	[− 3.57, − 3.38]	[− 2.07, − 2.00]	[− 0.84, − 0.79]	[0.87, 0.96]	[2.00, 2.17]	[− 0.11, − 0.07]
Number of left-censored persons in sample (by birth decade)				659	3687	4408	485	102	
Percentage of all persons in sample (by birth decade)				38.8%	23.9%	8.5%	0.8%	0.4%	
Including left-censored persons	Only cohort-effect	5.774	–	1.138	1.101	0.759	− 0.729	− 0.977	–
		[5.67, 5.88]		[1.08, 1.20]	[1.07, 1.13]	[0.74, 0.78]	[− 0.77, − 0.69]	[− 1.05, − 0.90]	
	Only age-effect	0.433	0.137	–	–	–	–	–	–
		[0.42, 0.44]	[0.137, 137]						
	Definition 1	0.190	0.183	− 2.095	− 1.177	− 0.441	0.525	1.342	− 0.039
		[0.18, 0.20]	[0.181, 0.185]	[− 2.16, − 2.03]	[− 1.21, − 1.14]	[− 0.47, − 0.42]	[0.48, 0.57]	[1.26, 1.42]	[− 0.06, − 0.02]

95% Confidence intervals based normality of Theorem 1 with standard errors from inverse of (9)

The preliminary model with only a cohort-effect has a likelihood (and (5)) with one factor for each cohort. Such stratified analysis simply fits each cohort to a separate (one-dimensional) Exponential distribution. With or without left-censoring, the right-censored data sets do not pose any numerical obstacles. The point estimates for excluded (top) and included (bottom) left-censored persons (Table 3, first rows) generally suggest a decrease in dementia hazard over the cohorts. The effect of including left-censoring is that the hazard rate is increased for all cohorts. Of course this is expected, because excluding right-censored observations is known to overestimate the hazard, and hence excluding left-censored observations should underestimate the hazard. The increase in hazard over the cohorts is not constant and we will observe and soon explain this phenomenon in the model of Definition 1. For the twentieth century’s first decade, the hazard itself is $\exp (1.138) \approx 3$ (with left-censored observations), i.e. it is three times higher then in the 1930s. In the most recent cohort of the 1950s, the hazard is $\exp (-0.977)\approx 0.4$, i.e. only 40% of the risk that prevails in the 1930s. We will soon see that this remarkable range can be explained (in part) with the model of Definition 1.

For the preliminary model with only an age-effect, we need to maximize (5) in two parameters, being a slightly larger numerical effort, because a visual inspection of (5) no longer suffices. Estimates are given in the second rows of Table 3 and the effect of $\beta _1 \approx 0.14$ (again with left-censored observations) means that with each additional year (starting at age 50), the dementia hazard (which is approximately the probability acquiring dementia within one year), is multiplied by $\exp (0.137) \approx 1.15$, i.e. by 15%. Again, excluding left-censoring decreases the parameter ${\hat{a}}$, here by a remarkable 50%.

The results of the Cox-type model in the third rows are similar to the next rows of Definition 1’s results, only with slight increases in standard errors due to accounting for the increased insecurity by the nonparametric baseline. This indicates robustness or admissibility of the Gompertz-assumption in Definition 1. The point is further verified by plotting the Breslow-estimate of the cumulative nonparametric baseline hazard in the Cox-type model and one finds that (for different resolutions) the (double-)exponential increase in age fits.

Let us now come to the model of Definition 1 with estimates given in the forth rows of Table 3. We discuss the cohort effect $\hat{\varvec{\beta }}_2$, the age effect ${\hat{\beta }}_1$ and the sex effect ${\hat{\beta }}_3$.

Let us start with the cohort effect and with a finding known as Simpson’s paradox. The ‘slope’ of the cohort effect is reverted, in comparison with the preliminary model with only the cohort effect. The sign of a linear approximation through $\beta _{21}, \ldots , \beta _{25}$ was preliminarily positive and is now negative. As an example, for a person born between 1900 and 1910, the dementia hazard is estimated as $\exp (-2.095)\approx 0.12$, i.e. is only 12% of those born in the 1930’s (with left-censored observations). By contrast, for a person from the 1950s, the hazard is $\exp (1.342) \approx 4$ and hence almost four times as high. As an interpretation, apparently, estimating the preliminary model with cohort effects only, in addition to a constant age-independent hazard which identifies the 1930 cohort, attributes the entire age effect of dementia incidence to the cohorts. Obviously, in the more recent cohorts, we exclusively observe lower ages, and we wrongly attribute the age effect to that cohort. By doing so, we greatly overestimate the decline in dementia incidence over cohorts.

A consequence of including left-censored persons, already remarked upon in the preliminary model with only the cohort effect, is that the slope in the cohort effect declines (in absolute terms). The reason why the hazard-increasing inclusion of left-censoring is not constant across cohorts is as follows. As can be seen from two rows in Table 3, the percentages of left-censored persons differ between the birth cohorts (i.e. within $\tilde{{\mathbf {z}}}$). In older, earlier cohorts, the portion is larger than in younger, more recent cohorts. As a result, in the older cohorts, the hazard estimate increases more than in younger cohorts, and the slope of such a cohort effect in $\varvec{\beta }_2$ becomes smaller. (The overall hazard increase is absorbed in the parameters *a* and $\beta _1$.)

**Fig. 5:**
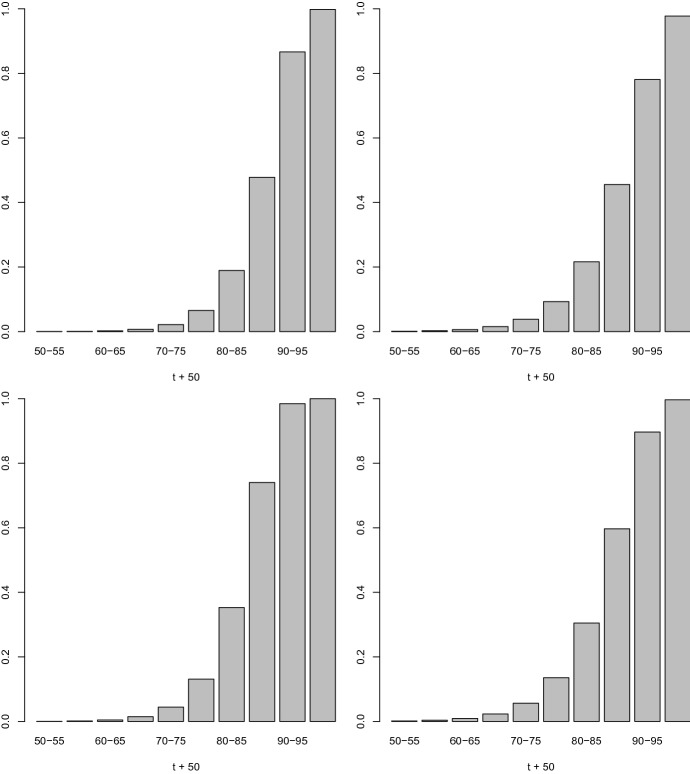
Five-year dementia incidence rates (top) male, born in 1920s, (bottom) female, born in 1930s—excluding left-censored persons (left), including left-censored persons (right)—for t + 50 = 50, 55, 60, ..., 95 year olds

Let us study the impact of left-censored persons in the model of Definition 1 on the age effect. First note that by including left-censored persons, ${\hat{a}}$, i.e. of the hazard for a 50-year-old male person born in the 1930s, rises, as to be expected. More interesting than the increase of ${\hat{a}}$ from 0.058 to 0.190 is the dip of ${\hat{\beta }}_1$ from 0.226 to 0.183. That means the increase in hazard is stronger for younger people compared to older ones. The impact becomes evident with the five-year incidence rates calculated from the conditional CDF (1), $P(Y^{\star } \in [t, t+d)| Y^{\star } \ge t) = [F(t+d|{\varvec{z}}) - F(t|{\varvec{z}})]/[1-F(t|{\varvec{z}})]$ ($d=5$), for a man born in 1920s, i.e. for ${{\tilde{z}}}_3 = 1, z^s = 0$ (for $t=0,5,10, \ldots 45$ in Fig. 5, top) and a woman born in 1930s, coded by ${{\tilde{z}}}_j \equiv 0$ and $z^s = 1$ (bottom). Including left-censored persons seems to produce a general dip in the hazard function, but the hazard really increases for younger people and decreases for older ones.

The statistical reason we advocated in the cohort effect, namely that more persons in earlier cohorts are left-censored, so that including them increases the estimate more than for later cohorts, is not applicable here. To the contrary, the fact that more persons are left-censored in earlier cohorts, now means that fewer are left-censored at lower ages. We cannot comment on the effect without a degree of speculation. Assume that (i) an older person, say born before 1930, belonging to the data, i.e. alive in 2004, is an indication of a health privilege. (Younger persons in the data who were been born after 1930 are not indicated.) Hence, the proportion of health-privileged among the elderly in the data must be disproportional. Assume further that (ii) a left-censored person has had dementia incidence early in life, and therefore cannot be regarded as health-privileged. As a consequence of both assumptions, including the left-censored ones, will reduce the disproportion of privileged and the age-effect must dip. Inference regarding the topic would require including further covariates in the model, observed or unobserved.

The consequence of including left-censored persons for the sex effect is an increase. The negative sex-effect, favourable to women, becomes smaller (in absolute terms) being ultimately almost irrelevant.

### Inference to demographic population

**Fig. 6:**
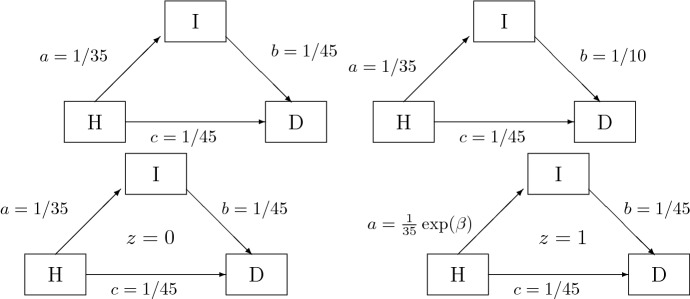
Three possible models: with equal death intensities (top, left), with unequal death intensities (top, right), with cohort/age effect (bottom) (H healthy, I ill, D dead)

A next step is to consider the statistical population as random sample of the demographic population (see Fig. 1). We will refrain here to comment on the effect of the 2nd stage selection of persons to the specific health insurance. However, we consider 1st stage selection of left-truncating those not surviving 2003. We will see that, for the morbidity analysis, left-censoring is a competing concept to left-truncation. For studying measurements such as mortality causing absorption, the case is different. Including measurements that are not absorbed, but contain only left-censored information, must be better than ignoring them combined with a general adjustment for left-truncation. In order to precisely discriminate between truncation due to early dementia and truncation due to prior death, we must include another state in the healthy-ill model and advance to a multi-state model, namely a healthy-ill-dead model (see Fig. 6(any panel)). In a Markovian model left-censoring, with respect to the illness, can be combined with left-truncation, caused by death.

An analytic treatment of the multi-state model, however without left-censoring and left-truncation, is found e.g. in Weißbach and Walter (2010); Kim et al. (2012). For an introduction to truncation see Weißbach et al. (2013); Frank et al. (2019); Dörre and Emura (2019). Here we simulate (i) homogeneous Markov processes, i.e. without time-effect (see Fig. 6(top)) and (ii) an inhomogeneous Markov process, i.e. with time-effect which will be a cohort-effect (simplified here to two intervals, $z=0$ and $z=1$) (see Fig. 6(bottom)). A homogeneous Markov process can be easily simulated by its construction of exponential waiting times and target states with multinomial distribution (Albert 1962, see e.g.). An inhomogeneous process is simulated as easily.

We assume births, i.e. the 50th birthday, to be uniformly distributed on [1950, 2004]. The time-homogeneous, i.e. age- and cohort-constant, transition intensities are $a = 1/35$ (in the notation of Definition 1), $b=1/45$ and $c=1/45$ and resemble the data set. Durations are right-censored either by death or end of data collection (at the beginning of 2014). A person is omitted by left-truncation if it does not survive 2003.

All simulation results are based on 10,000 repetitions of the same set-up. For all models, the transition intensity from healthy to ill is the hazard rate and estimated with (3). (Fitting all parameters to the data would be based on the partial likelihood of the multiple Markov process (see Andersen et al. 1993, equation 2.7.4’), however including left-censoring is not straight-forward.) The Bias of any estimator ${\hat{\gamma }}$ is calculated as $\mathrm {B(IAS)}({\hat{\gamma }}) := \bar{{\hat{\gamma }}} - \gamma$ and the relative Bias as $\mathrm {rB(IAS)}({\hat{\gamma }}) := 100 \cdot \mathrm {B(IAS)}({\hat{\gamma }})/\gamma$. Recall from Fig. 1 that *N* denotes the size of the demographic population, before 1st stage selection, i.e. left-truncation, and *n* the (random) sample size (after truncation and possibly after removing left-censored persons). The number of left-truncated persons is denoted by $n_{LT}$ and of left-censored persons by $n_{LC}$.

First, ignoring left-truncation does not necessarily bias the illness incidence estimation, if left-censoring is taken into account (see Fig. 6(top, left)). Table 4 shows the negative bias for ignoring both and a negligible bias when accounting for left-censoring.

**Table 4:** Left-truncation ignored, right-censoring considered

*N*	*E*(*n*)	$E(n_{LT})$	$E(n_{LC})$	$\mathrm {BIAS}(a_{HI})$	$\mathrm {rBIAS}(a_{HI})$
Left-censoring ignored					
100	34.05	41.80	24.15	$-$ 0.01905	$-$ 66.69
250	85.30	104.43	60.27	$-$ 0.01911	$-$ 66.90
1000	340.90	417.78	241.32	$-$ 0.01913	$-$ 66.94
Left-censoring considered					
100	58.29	41.71	24.11	0.000369	1.29
250	145.47	104.53	60.27	0.000166	0.58
1000	582.30	417.70	241.19	0.000055	0.19

Second, that the statistical population is not a simple sample, i.e. with equal selection probabilities, becomes visible, when assuming cohort effects. Persons of an earlier cohort have a smaller probability to survive 2003 than later cohorts. Hence it is questionable whether the cohort effect $\varvec{\beta }_2$ of Definition 1 is estimated correctly. In order to account for a cohort effect (see Fig. 6 (bottom)), define for cohort 1950–1977 $z=0$ with hazard $a(t|0) \equiv 1/35$, i.e. with $a=1/35$, and for cohort 1978–2003 $z=1$ with hazard $a(t|1) = \frac{1}{35} \exp (\beta )$ and $\beta =0.7$. Again, truncation due to death does introduce a bias into the estimate of the cohort effect (see Table 5(top)). However, the cohort effect is apparently estimated consistently, at least for age-constant intensities, if left-censoring is accounted for (see Table 5(bottom)).

**Table 5:** Multiplicative cohort effect, left-truncation ignored, right-censoring considered

*N*	*E*(*n*)	$E(n_{LT})$	$E(n_{LC})$	$\mathrm {B}(a_{HI})$	$\mathrm {B}(\beta )$	$\mathrm {rBI}(a_{HI})$	$\mathrm {rBI}(\beta )$
Left-censoring ignored							
100	27.8	41.6	30.6	$-$ 0.02290	1.061	$-$ 80.15	151.5
250	69.2	104.5	76.3	$-$ 0.02339	0.985	$-$ 81.85	140.7
1000	277.2	417.9	304.9	$-$ 0.02258	0.879	$-$ 79.02	125.5
Left-censoring considered							
100	58.2	41.8	30.6	0.00161	$-$ 0.00803	5.63	$-$ 1.15
250	145.6	104.4	76.4	0.00048	0.00191	1.55	$-$ 0.27
1000	582.5	417.5	305.6	0.00006	0.00190	0.22	0.27

Third, one should not have the impression that left-censoring can replace left-truncation in any situation. If the death intensity is different for the transition from healthy to dead *c* as compared to the transition from ill to dead *b* (see Fig. 6(top, right)), a (small) bias is introduced by ignoring the left-truncation phenomenon even if one accounts for left-censoring (see Table 6).

With respect to standard errors for the inference to the demographic population, note that $E_{{\mathbf {Z}}}$ of Theorem 1 will still have a proper meaning, however, (9) will not necessarily estimate the asymptotic variance. One reason is that the covariate ${\mathbf {Z}}$ is not random but deterministic from the demographic population to $1^{st}$ stage selection (see Fig. 1), another theory must be applied (see e.g. Bradley and Gart 1962; Weißbach and Radloff 2020).

**Table 6:** Left-truncation ignored, left-censoring and right-censoring considered, varying mortality

*N*	*n*	$E(n_{LT})$	$E(n_{LC})$	$\mathrm {BIAS}(a_{HI})$	$\mathrm {rBIAS}(a_{HI})$
100	43.2	56.8	9.1	$-$ 0.00991	$-$ 34.7
250	108.0	142.1	22.7	$-$ 0.01005	$-$ 35.2
1000	431.9	568.1	91.0	$-$ 0.0101	$-$ 35.3

## Conclusion

The study reveals that even when including left-censored observation in a survival analysis, the asymptotic analysis of the model may use elementary means. However, bypassing lengthy calculations, as in Kremer et al. (2014) for left-censored observation only, is no longer possible. Only with right-censoring, would the model be a member of the exponential family (and also a generalized linear model). Whether double-censoring can be analysed more easily in a counting process framework, was not investigated. As are the additional conditions to (6) for consistency. Also, together with the major assumption of estimator consistency, there are also further assumptions, such as the existence of $\varvec{{\mathcal {J}}}^{-1}$, needing investigation in order to conclude asymptotically normality estimator (see e.g Theorem 5.21 in van der Vaart 1998).

An application to issues of human morbidity in follow-up studies is appealing, because a disease typically does not ‘absorb’ the statistical unit. And due to the longevity of humans, many follow-up studies typically cannot start before to the first disease incidence. In particular here, left-censoring accounts for the general weakness of the HCD that it one does not follow each cohort from a given age, but rather for a given period.

From a broader perspective, estimating the duration distribution conditional only on survival may be unsatisfactory. However, note that the probability of an incidence exceeding age *t*, given the lifetime surpassed age *s*, can be multiplied with the last probability, so as to result in the joint distribution. In order to obtain the marginal distribution of the disease incidence, only the second argument needs to be integrated out. Hence, because the mortality distribution will typically be known quite accurately from other data sources such as a register of deaths, knowing the conditional distribution is already a major achievement.

An unconditional estimate, solely from the HCD, will first have to reconsider the assumption in Definition 1, that the dummy variable coding the cohort, e.g. ${\tilde{Z}}_4$ for the 1940s (see again Table 1), is endogenous for the demographic population. Its parameter is the probability of selecting a person born during that decade in the data process of Fig. 1. This probability is dependent on the probability of surviving 2003. If the probability of surviving depends on having dementia (which is widely accepted), the probability of dementia incidence (and hence the model of Definition 1 and its parameter $\varvec{\theta }$) will be influential.

## Appendix A: Proofs for Sect. 4.1

### Lemma 4

There $\not \exists (y^{\star }, a_0)$, $y^{\star } \in \mathbb {R}^+, a_0 \in \Theta$, such that $lr_{a_0,y^{\star }}(a):= \log (a/a_0) - (a - a_0) y^{\star } < 0$ as a function of *a*, i.e $\forall \; a \in \Theta$.

### Proof

Assume the converse. The function $lr_{a_0,y^{\star }}(a)$ has its maximum with respect to *a* in $1/y^{\star }$, so negativity is equivalent to

A.1 $$\begin{aligned} \log \frac{1}{y^{\star } a_0} - \left( \frac{1}{y^{\star }} - a_0\right) y^{\star }< 0 \Leftrightarrow q_{a_0}(y^{\star }):= - \log (y^{\star } a_0) + y^{\star } a_0 < 1. \end{aligned}$$

Function $q_{a_0}(\cdot )$ is convex as its second derivative is $1/y^{\star 2}>0$. (Note that, being in survival analysis, we may refrain from allowing $y^{\star }=0$.) The first derivative of $q_{a_0}(\cdot )$ is zero for $y^{\star }=1/a_0$, being the minimum. At the minimum of $q_{a_0}(\cdot )$ is

$$\begin{aligned} q_{a_0}\left( \frac{1}{a_0}\right) = - \log \left( \frac{1}{a_0} a_0 \right) + a_0 \frac{1}{a_0} = 1 \not < 1. \end{aligned}$$

Hence it is $\ge 1$ for any $a_0$, and there exists no $a_0$ to fulfil (A.1), being a contradiction. $\square$

Before we continue the proof of Lemma 1, we note some useful properties:

A.2 $$\begin{aligned} \begin{array}{ccccc} \hline \varepsilon< a_0 &{} \Leftrightarrow &{} \frac{\varepsilon }{a_0}<1 &{} \Leftrightarrow &{} \log \frac{\varepsilon }{a_0}<0 \\ a_0< \frac{1}{\varepsilon } &{} \Leftrightarrow &{} 1< \frac{1}{\varepsilon a_0} &{} \Leftrightarrow &{} \log \frac{1}{\varepsilon a_0}> 0 \\ a_0< \frac{1}{\varepsilon } &{} \Leftrightarrow &{} \varepsilon a_0 < 1 &{} \Leftrightarrow &{} 1 - \varepsilon a_0 > 0 \\ \hline \end{array} \end{aligned}$$

Inserting $\varepsilon$, $1/y^{\star }$ or $1/\varepsilon$ into log likelihood ratio (7) as *a*) bounds it in absolute terms. Of course relevant candidate can vary for different $y^{\star }$. We denote the potential bounds by $g_{\varepsilon }^{abs}(y^{\star })$, $g_{\frac{1}{y^{\star }}}^{abs}(y^{\star })$ and $g_{\frac{1}{\varepsilon }}^{abs}(y^{\star })$ and give the definition in the following table. It contains also simplified versions thereof, useful as we see soon.

$$\begin{aligned} \begin{array}{ccc} \hline g_{\varepsilon }(y^{\star }) &{} := &{} \log \frac{\varepsilon }{a_0} - (\varepsilon - a_0) y^{\star } \\ g_{\varepsilon }^{abs}(y^{\star }) &{} := &{} \left| \log \frac{\varepsilon }{a_0} - (\varepsilon - a_0) y^{\star } \right| \\ \hline g_{\frac{1}{y^{\star }}}(y^{\star }) &{} := &{} \log \frac{1}{y^{\star } a_0} - (\frac{1}{y^{\star }} - a_0) y^{\star } \\ g_{\frac{1}{y^{\star }}}^{abs}(y^{\star }) &{} := &{} \left| \log \frac{1}{y^{\star } a_0} - (\frac{1}{y^{\star }} - a_0) y^{\star } \right| \\ \hline g_{\frac{1}{\varepsilon }}(y^{\star }) &{} := &{} \log \frac{1}{\varepsilon a_0} - (\frac{1}{\varepsilon } - a_0) y^{\star } \\ g_{\frac{1}{\varepsilon }}^{abs}(y^{\star }) &{} := &{} \left| \log \frac{1}{\varepsilon a_0} - (\frac{1}{\varepsilon } - a_0) y^{\star } \right| \\ \hline \end{array} \end{aligned}$$

Using (A.2), one sees that function $g_{\varepsilon }(\cdot )$ is linearly increasing, with negative $y^{\star }$-axis off-set, and function $g_{\frac{1}{\varepsilon }}(\cdot )$ is linearly decreasing, with positive $y^{\star }$-axis off-set.

Both, $g_{\varepsilon }^{abs}(\cdot )$ and $g_{\frac{1}{\varepsilon }}^{abs}(\cdot )$, are positive and linear functions with slopes $a_0 - \varepsilon$ and $1/\varepsilon - a_0$ from some point $y^{\star }$ on. (A linear function in the integration of (6) will result in an expectation, and hence be finite.)

Unfortunately this is not true for $g^{abs}_{\frac{1}{y^{\star }}}(\cdot )$ but the following will simplify the integration. Function $g_{\frac{1}{y^{\star }}}(\cdot )$ is convex, because $\frac{d^2}{d y^{\star 2}}g_{\frac{1}{y^{\star }}}(y^{\star })= \frac{1}{y^{\star 2}} > 0$. As $\frac{d}{d y^{\star }} g_{\frac{1}{y^{\star }}}(y^{\star })= a_0 - \frac{1}{y^{\star }}$ it is minimal in $y^{\star }=1/a_0$. Because $g_{\frac{1}{y^{\star }}}(\frac{1}{a_0}) = 0$ the function is non-negative and hence $g_{\frac{1}{y^{\star }}}(\cdot ) \equiv g^{abs}_{\frac{1}{y^{\star }}}(\cdot ) \equiv g^+_{\frac{1}{y^{\star }}}(\cdot )$.

This and using $\max (x,y) \le x + y$ (for positive *x* and *y*) we define

A.3 $$\begin{aligned} h(y^{\star }):= & {} \max \{g_{\varepsilon }^{abs}(y^{\star }),g_{\frac{1}{\varepsilon }}^{abs}(y^{\star }) \} + g_{\frac{1}{y^{\star }}}^{abs}(y^{\star }) \nonumber \\= & {} \max _{a \in \{\varepsilon , \frac{1}{\varepsilon }\}} \left| \log \frac{a}{a_0} - (a - a_0)y^{\star } \right| + \log \frac{1}{y^{\star } a_0} - \left( \frac{1}{y^{\star }} - a_0\right) y^{\star }. \end{aligned}$$

For the two bounding function candidates $g_{\varepsilon }^{abs}(0)$ and $g_{\frac{1}{\varepsilon }}^{abs}(0)$ are below positive $- \log \frac{\varepsilon }{a_0}$ or $\log \frac{1}{\varepsilon a_0}$ (see (A.2)).

Now

A.4 $$\begin{aligned}&\int _0^{\infty } h(y^{\star }) f_{a_0}(y^{\star })dy^{\star } \le \int _0^{\infty } \max \{g_{\varepsilon }^{abs}(y^{\star }),g_{\frac{1}{\varepsilon }}^{abs}(y^{\star }) \} f(y^{\star };a_0) dy^{\star } \nonumber \\&+ \int _0^{\infty } \log \frac{1}{y^{\star } a_0} - \left( 1 - a_0 y^{\star }\right) f(y^{\star };a_0) dy^{\star }. \end{aligned}$$

Both candidate functions $g_{\varepsilon }^{abs}(\cdot )$ and $g_{\frac{1}{\varepsilon }}^{abs}(\cdot )$ can be bounded from above by linear function $\max \{- \log \frac{\varepsilon }{a_0}, \log \frac{1}{\varepsilon a_0} \} + \max \{\varepsilon - a_0, \frac{1}{\varepsilon } - a_0 \} y^{\star }$. Hence the first integral on the right in (A.4) is smaller than

$$\begin{aligned} \max \left\{ - \log \frac{\varepsilon }{a_0}, \log \frac{1}{\varepsilon a_0} \right\} \int _0^{\infty } f(y^{\star };a_0) dy^{\star } + \max \{\varepsilon - a_0, \frac{1}{\varepsilon } - a_0 \} \int _0^{\infty } y^{\star }f(y^{\star };a_0) dy^{\star } \end{aligned}$$

and finite as the first integral is one and the second the expectation $1/a_0$.

For the second line in (A.4), with the above arguments, it suffices to show

$$\begin{aligned} \int _0^{\infty } - \log (y^{\star } a_0) f(y^{\star };a_0) dy^{\star }< \int _0^{\frac{1}{a_0}} - \log (y^{\star } a_0) f(y^{\star };a_0) dy^{\star } < \infty \end{aligned}$$

Furthermore wlog $a_0$ can be set to 1 here, i.e. the only term to ensure finiteness is

$$\begin{aligned} \lim _{y^{\star }_n \rightarrow 0} \int _{y^{\star }_n}^1 - log (y^{\star }) e^{-y^{\star }} dy^{\star }\le & {} \lim _{y^{\star }_n \rightarrow 0} \int _{y^{\star }_n}^1 - log (y^{\star }) dy^{\star } = \lim _{y^{\star }_n \rightarrow 0} - \left[ y^{\star } \log y^{\star } - y^{\star } \right] _{y^{\star }_n}^1 \\= & {} \lim _{y^{\star }_n \rightarrow 0} - \left[ \log 1 - 1 - y^{\star }_n \log y^{\star }_n + y^{\star }_n \right] = 1 + \lim _{y^{\star }_n \rightarrow 0} \frac{\log y^{\star }_n}{\frac{1}{y^{\star }_n}} \\= & {} 1 - \lim _{y^{\star }_n \rightarrow 0} \frac{\frac{1}{y^{\star }_n}}{\frac{1}{y^{\star 2}_n}} = 1 \end{aligned}$$

For the first equality, see Formula 4.1.49 in Abramowitz and Stegun (1970), the last but one equality follows from de l’Hôspital’s rule. This ends the proof of Lemma 1.

For the proof of Lemma 2 note first that

$$\begin{aligned} \Psi _L(y,\delta ;a)=(1+ \delta )(\log (a) - a y) - \delta \log (1 - e^{-a y}). \end{aligned}$$

The search for maxima of $|\Psi _L(y,\delta ;a)|$ wrt *a* for a fixed $(y,\delta )$ means that we need maxima for $|\Psi _L(y,0;a)|$ and $|\Psi _L(y,-1;a)|$, both for a fixed *y*. The maxima of the first are known from the proof of Lemma 1 to be in $\varepsilon$, 1/*y* and $1/\varepsilon$. Now look at the necessary condition

A.5 $$\begin{aligned} \int _{\mathbb {R}_+} h_L(y,0) f_{Y,\Delta }(y,0; a_0) dy + \int _{\mathbb {R}_+} h_L(y,-1) f_{Y,\Delta }(y,-1; a_0) dy < \infty . \end{aligned}$$

In the first summand the second factor is proportional to the density of $(Y,\Delta )$ conditional on $\Delta$. It only needs to be divided by one minus the probability of censoring, $P(Y^{\star }>L)$, which is by assumption smaller than one. The function $h_L(y,0)$ can now be chosen as *h*(*y*) in Lemma 1 and hence the summand is proportional to $E(Y^{\star })=1/a_0 < \infty$.

For the second summand in (A.5) define $g(a):= \log (1 - e^{- a y})$. The argument of the logarithm is a monotone CDF of an Exponential distribution, as a function of *a*. It starts in zero and ends in one and hence $g(a) <0$ and $\lim _{a \searrow 0}= - \infty$ and $\lim _{a \rightarrow \infty }= 0$. Hence

$$\begin{aligned} h_L(y,-1):= \max _{a \in [\varepsilon ,1/\varepsilon ]} |g(a)| = - g(\varepsilon )= - \log (1 - e^{- \varepsilon y}) \end{aligned}$$

and the second summand in (A.5) is

A.6 $$\begin{aligned} \int _{\mathbb {R}^+} - \log (1 - e^{- \varepsilon y}) (1-e^{a_0 y}) f_L(y) dy \end{aligned}$$

as $f_{Y,\Delta }(y,-1; a_0)$ is $(1-e^{a_0 y}) f_L(y)$ due to (3). We will now discuss why the integrand (without $f_L(y)$) is bounded so that the integral is finite. For $y \rightarrow \infty$ we have

$$\begin{aligned} {\tilde{h}}_L(y):=- \log (1 - e^{- \varepsilon y}) (1-e^{a_0 y}) \end{aligned}$$

becomes zero, because $(1-e^{a_0 y})$ converges to one and $- \log (1 - e^{- \varepsilon y})$ to zero. It is

$$\begin{aligned} \lim _{y \searrow 0} \underbrace{- \log (1 - e^{- \varepsilon y})}_{\rightarrow + \infty } \underbrace{(1-e^{a_0 y})}_{\rightarrow 0} = \lim _{y \searrow 0} \frac{- \log (1 - e^{- \varepsilon y})}{(1-e^{a_0 y})^{-1}}= 0, \end{aligned}$$

as can be seen by two times applying l’Hôspital’s rule. Note the ${\tilde{h}}_L(y)>0$ and is zero on both edges of its support $\mathbb {R}^+=(0, \infty )$. It is continuous on $[0, \infty ]$, as a product of continuous functions on $\mathbb {R}^+$, where the first is continuous as a composition of continuous functions. By the mean value theorem there must be a *y* with ${\tilde{h}}'_L(y)=0$, being a maximum. Even if we do not show here uniqueness, as a continuous function on the compact $[\varepsilon _y, 1/\varepsilon _y]$ for sufficiently small $\varepsilon _y$, it must attain its maximum. Without poles on $[0, \infty ]$ this must be a finite *M*. The density $f_L(y)$ now bounds (A.6) with *M* and ends the proof of Lemma 2.

## Appendix B: Proof for Sect. 4.2

For the log likelihood it is well-known that the expected squared first derivative and the expected negative second derivative of the log likelihood are equal (see e.g. Formula (5.36) of van der Vaart 1998), alike for the CMLE (see Formula (13.27) of Wooldridge 2010 or Sec. 7.2.2 of McCullagh and Nelder 1989). Lemma 3 disposes of the covariate parameters and finally of $\varvec{\theta }_{\Delta }$, in order to state the similar result for the ‘upper-left’ $8 \times 8$-block matrix.

To conclude $\varvec{{\mathcal {I}}} = \varvec{{\mathcal {J}}}$ in more detail denote the logarithmic conditional density for person *i* by $L_i(\varvec{\theta },\varvec{\theta }_{\Delta }):= L[(Y_i, \Delta _i),{\mathbf {Z}}_i]:=\log f_{Y,\Delta |{\mathbf {Z}}}(Y_i, \Delta _i|{\mathbf {Z}}_i)$ from (3), only with additional right-censored observations and generic $\varvec{\theta }$. Now define ${\mathbf {s}}_i(\varvec{\theta }):=\nabla _{\varvec{\theta }} L_i(\varvec{\theta },\varvec{\theta }_{\Delta })=\nabla _{\varvec{\theta }} \Psi [(Y_i, \Delta _i), {\mathbf {z}}_i; \varvec{\theta }]$ as vector of eight partial derivatives, as the factors of (3) belonging to censoring will vanish after taking logarithm and differentiation.

### Lemma 5

It holds $E_0[{\mathbf {s}}_i(\varvec{\theta }_0)]={\mathbf {0}}$, and hence unconditionally $E_{{\mathbf {Z}}}\{E_0[{\mathbf {s}}_i(\varvec{\theta }_0)]\}={\mathbf {0}}$.

### Proof

Denote by $E_{\varvec{\theta },\varvec{\theta }_{\Delta }}( \cdot |{\mathbf {Z}}_i={\mathbf {z}}_i)$ the conditional expectation with respect to density (3) (again with right-censoring), which is similar to $E_0$, however, with generic $(\varvec{\theta },\varvec{\theta }_{\Delta })$. It is

B.1 $$\begin{aligned} E_{\varvec{\theta },\varvec{\theta }_{\Delta }}({\mathbf {s}}_i(\varvec{\theta })|{\mathbf {Z}}_i={\mathbf {z}}_i) = \int _{\mathbb {R}^+} \sum _{\delta \in \{-1,0,1\}} {\mathbf {s}}_i[(y, \delta ), {\mathbf {z}}_i, \varvec{\theta }] f_{Y,\Delta |{\mathbf {Z}}}(y, \delta |{\mathbf {z}}_i) dy. \end{aligned}$$

Due to the chain rule for differentiation, applied to the logarithm in $L_i(\varvec{\theta },\varvec{\theta }_{\Delta })$, it is

B.2 $$\begin{aligned} \nabla _{\varvec{\theta }} f_{Y,\Delta |{\mathbf {Z}}}(y_i, \delta _i|{\mathbf {z}}_i) = \left\{ \nabla _{\varvec{\theta }} L[(y_i, \delta _i),{\mathbf {z}}_i]\right\} f_{Y,\Delta |{\mathbf {Z}}}(y_i, \delta _i|{\mathbf {z}}_i). \end{aligned}$$

Integrating wrt $y_i$ and summation wrt $\delta _i$ results on the left-hand side in zero, because after interchanging differentiation $\nabla _{\varvec{\theta }}$ with integration and summation, what needs to be taken a derivative of, is one, due to the density property. On the right-hand side, recall the definition of ${\mathbf {s}}_i(\varvec{\theta })$. Now set $\varvec{\theta }_0$ for $\varvec{\theta }$. $\square$

As $L_i(\varvec{\theta },\varvec{\theta }_{\Delta })$ is twice differentiable wrt $\varvec{\theta }$, let the Hessian for observation *i* denote

$$\begin{aligned} {\mathbf {H}}_i(\varvec{\theta }):= \nabla _{\varvec{\theta }'} {\mathbf {s}}_i(\varvec{\theta }) = \nabla ^2_{\varvec{\theta }} L_i(\varvec{\theta },\varvec{\theta }_{\Delta }), \end{aligned}$$

where $\nabla _{\varvec{\theta }'}$ leads to a row vector (for each coordinate of the column vector to be taken the derivative of). It is obviously $\varvec{{\mathcal {J}}}=- E_{{\mathbf {Z}}} E_0[{\mathbf {H}}_i(\varvec{\theta }_0)]$ and we are now ready to show $- E_{{\mathbf {Z}}} E_0[{\mathbf {H}}_i(\varvec{\theta }_0)]= \varvec{{\mathcal {I}}}$. Because of the product rule for differentiation—generalized to vector coordinates—it is

B.3 $$\begin{aligned}&\nabla _{\varvec{\theta }'} [ {\mathbf {s}}\left[ (y_i, \delta _i), {\mathbf {z}}_i, \varvec{\theta }\right] f_{Y,\Delta |{\mathbf {Z}}}(y_i, \delta _i|{\mathbf {z}}_i) ] \nonumber \\&\quad =\nabla _{\varvec{\theta }'} {\mathbf {s}}\left[ (y_i, \delta _i), {\mathbf {z}}_i, \varvec{\theta }\right] f_{Y,\Delta |{\mathbf {Z}}}(y_i, \delta _i|{\mathbf {z}}_i) + {\mathbf {s}}\left[ (y_i, \delta _i), {\mathbf {z}}_i, \varvec{\theta }\right] \nabla _{\varvec{\theta }'} f_{Y,\Delta |{\mathbf {Z}}}(y_i, \delta _i|{\mathbf {z}}_i).\nonumber \\ \end{aligned}$$

Again applying $\int _{\mathbb {R}^+} \sum _{\delta \in \{-1,0,1\}}$ to the left-hand side and interchanging with $\nabla _{\varvec{\theta }'}$ results in a zero to be taken the derivative of (due to the arguments after (B.1)). When integrating the the right-hand side, replace in the second summand $\nabla _{\varvec{\theta }'} f_{Y,\Delta |{\mathbf {Z}}}(y_i, \delta _i|{\mathbf {z}}_i)$ with the transpose of (B.2). so that

$$\begin{aligned} - E_{\varvec{\theta },\varvec{\theta }_{\Delta }}\left[ {\mathbf {H}}_i(\varvec{\theta })|{\mathbf {Z}}_i={\mathbf {z}}_i\right] = E_{\varvec{\theta },\varvec{\theta }_{\Delta }}\left( {\mathbf {s}}_i(\varvec{\theta }){\mathbf {s}}_i(\varvec{\theta })'|{\mathbf {Z}}_i={\mathbf {z}}_i \right) . \end{aligned}$$

Inserting $\varvec{\theta }_0$, we have the conditional information matrix equality

$$\begin{aligned} - E_0[{\mathbf {H}}_i(\varvec{\theta }_0)] =E_0[{\mathbf {s}}_i(\varvec{\theta }_0) {\mathbf {s}}_i(\varvec{\theta }_0)']= E_0 \left( \frac{\partial \Psi [(Y, \Delta ), {\mathbf {Z}}; \varvec{\theta }_0]}{\partial \varvec{\theta } }\frac{\partial \Psi [(Y,\Delta ), {\mathbf {Z}}; \varvec{\theta }_0]}{\partial \varvec{\theta }' } \right) , \end{aligned}$$

because, again, $L_i$ and $\Psi$ have equal derivatives. Then, we take $E_{{\mathbf {Z}}}$ on both sides and use the iterated expectation theorem. This ends the proof of Lemma 3.
